# Revisiting the Interaction of Melittin with Phospholipid Bilayers: The Effects of Concentration and Ionic Strength

**DOI:** 10.3390/ijms21030746

**Published:** 2020-01-23

**Authors:** Thiru Sabapathy, Evelyne Deplazes, Ricardo L. Mancera

**Affiliations:** 1 School of Pharmacy and Biomedical Sciences, Curtin Health Innovation Research Institute, Curtin University, GPO Box U1987, Perth, WA 6845, Australia; t.sabapathy@curtin.edu.au (T.S.); evelyne.deplazes@uts.edu.au (E.D.); 2 School of Life Sciences, University of Technology Sydney, Ultimo, NSW 2007, Australia

**Keywords:** surface plasmon resonance, melittin, liposomes, peptide–lipid interactions, anti-microbial peptides, pore-forming peptides

## Abstract

Melittin is an anti-microbial peptide (AMP) and one of the most studied membrane-disrupting peptides. There is, however, a lack of accurate measurements of the concentration-dependent kinetics and affinity of binding of melittin to phospholipid membranes. In this study, we used surface plasmon resonance spectroscopy to determine the concentration-dependent effect on the binding of melittin to 1-palmitoyl-2-oleoyl-glycero-3-phosphocholine (POPC) bilayers in vesicles. Three concentration ranges were considered, and when combined, covered two orders of magnitudes (0.04 µM to 8 µM), corresponding to concentrations relevant to the membrane-disrupting and anti-microbial activities of melittin. Binding kinetics data were analysed using a 1:1 Langmuir-binding model and a two-state reaction model. Using in-depth quantitative analysis, we characterised the effect of peptide concentration, the addition of NaCl at physiological ionic strength and the choice of kinetic binding model on the reliability of the calculated kinetics and affinity of binding parameters. The apparent binding affinity of melittin for POPC bilayers was observed to decrease with increasing peptide/lipid (P/L) ratio, primarily due to the marked decrease in the association rate. At all concentration ranges, the two-state reaction model provided a better fit to the data and, thus, a more reliable estimate of binding affinity. Addition of NaCl significantly reduced the signal response during the association phase; however, no substantial effect on the binding affinity of melittin to the POPC bilayers was observed. These findings based on POPC bilayers could have important implications for our understanding of the mechanism of action of melittin on more complex model cell membranes of higher physiological relevance.

## 1. Introduction

Anti-microbial peptides (AMPs) are found throughout the animal and plant kingdom, where they form an important part of the innate immune system [1]. Due to the fact of their potent activity against Gram-positive and Gram-negative bacteria as well as other pathogens, AMPs have been actively pursued as lead molecules for the development of new antimicrobial agents [2,3,4,5]. In addition, AMPs have been investigated for their anti-cancer activity to address the issue of chemotherapy resistance [6,7,8], and there is emerging evidence for their use as adjuvant cancer therapeutics [9,10].

The cytotoxic effect of many AMPs primarily stems from their ability to lyse the phospholipid bilayer of the plasma or mitochondrial cell membranes [11,12,13]. While AMPs show a wide range of secondary structures and different mechanisms of action, they share certain physico-chemical characteristics. Like other membrane-disruptive peptides, AMPs are positively charged (cationic) and have a relatively large number of hydrophobic residues that are often clustered to give the peptide an overall amphipathic character [5,11,12,13,14,15]. Their cationic nature enables electrostatic interactions with the negatively charged (anionic) lipid head groups found in bacterial membranes, while the amphipathic nature facilitates insertion into the hydrophobic core of the membrane. However, there is still an incomplete understanding of the molecular mechanisms by which AMPs bind to and, subsequently, disrupt cell membranes, as well as the physico-chemical factors affecting the interaction [12]. Characterising the membrane-binding properties of AMPs is particularly important for the development of therapeutic peptides (e.g., antibiotics or anti-cancer agents) with higher specificity for microbes or cancer cells.

One of the most studied AMPs is melittin [16], the major component of honey bee venom [17]. Melittin is a 26 amino acid long cationic peptide and its cytolytic effects on lipid vesicles as well as on bacterial and mammalian cells has been demonstrated in a large number of studies [18,19,20,21,22,23,24,25,26,27]. Early work using nuclear magnetic resonance (NMR) and circular dichroism (CD) spectroscopy on the structure of melittin in water and lipid environments showed that the peptide is mostly unstructured in solution but undergoes a transition to α-helix upon binding to a lipid–water or hydrophobic interface [28,29,30,31,32,33,34,35,36]. Melittin is also known to dimerise and form higher-order oligomers under certain conditions such as high peptide concentrations and high ionic strength [29,37,38,39].

The membrane-disrupting mechanism of melittin has been studied extensively using a wide range of experimental and molecular simulation approaches [40]. Findings from these studies suggest that membrane-bound melittin can be present in two orientations: parallel, where it lies on the membrane surface interacting mostly with the lipid head groups, and perpendicular, where it is inserted into the hydrophobic core of the membrane, oriented perpendicularly to the membrane surface. A two-step model is often used to describe the mechanism of formation of membrane pores by melittin [26,41,42], wherein the peptide, at low concentration, binds to the bilayer surface in a parallel conformation and then shifts to a perpendicular orientation at higher concentration, leading to membrane pore formation. The existence of two membrane-bound orientations has been demonstrated by molecular simulation studies [43,44,45,46,47,48] and the retention of amphiphilic peptides in a surface-absorbed state has been explained by elasticity theory [49].

A recent study has suggested a variation to this two-step model, in which the transition from parallel to perpendicular does not occur but, rather, parallel surface binding and direct insertion are competing processes [26]. Numerous studies of melittin make it clear that surface binding, membrane disruption and pore formation constitute an inter-dependent and complex process. In addition, each one of these processes is affected by environmental factors such as membrane lipid composition [23,27,39,50,51,52,53], peptide concentration and peptide–lipid ratio [21,54], pH and ionic strength [51,53], and local membrane curvature [55].

Like other AMPs, melittin exhibits preferred binding to negatively charged (anionic) lipids [53,56] due to the strong electrostatic attraction between the C-terminal region of melittin and negatively charged lipid headgroups [53]. It has been suggested that the basis for the strong attraction between melittin and anionic lipids is an “electrostatic arrest” (adsorption) of melittin in its parallel (inactive) orientation on the lipid surface [53]. Indeed, this postulate is used to explain the tolerance of anionic liposomes to membrane disruption by melittin [32,57,58]. This suggests that, compared to neutral (or zwitterionic membranes), the presence of anionic lipids promotes increased surface adsorption but, by favouring the (inactive) parallel conformation of melittin, it also hinders pore formation and, thus, reduces leakage. Likewise, melittin interacts with the headgroups of zwitterionic lipids (e.g., phosphatidylcholine), but the interaction is much weaker than for anionic lipids (e.g., phosphatidylglycerol or phosphatidylinositol) [53,55,59,60].

Among the most commonly used methods to study membrane binding of AMPs, including melittin, are isothermal titration calorimetry (ITC) [61] and surface plasmon resonance (SPR) [62,63,64,65]. Other methods used for the study of melittin to lipid membranes include ultrafiltration assays [55,59] and fluorescence measurements [53]. Isothermal titration calorimetry has been used to determine the enthalpy (ΔH), entropy (ΔS) and free energy of binding (ΔG) as well as the associated equilibrium dissociation constant (*K*_D_) of the interaction between melittin and model cell membranes with various lipid compositions [66,67]. Surface plasmon resonance has provided real-time measurements of the association (*k*_a_) and dissociation rates (*k*_d_) of binding as well as estimates of ΔG for melittin–lipid interactions [62,63,65,68]. In one of the earliest SPR studies, Aguilar and co-workers [64] reported on the binding of melittin to dimyristoylphosphocholine (DMPC) and dimyristoylphosphatidylglycerol (DMPG) using peptide concentrations between 10 and 140 μM. While this concentration range is not high enough to induce the formation of the tetrameric aggregate of melittin in solution [28,29,69,70,71], it may likely cause the accumulation of the peptide on the membrane surface thus affecting both the subsequent binding of melittin and the barrier properties of the lipid bilayer [72]. This is important because, based on the classical carpet model [73], it has been postulated that, at high concentration, cationic amphiphilic peptides (including melittin) can accumulate at the lipid surface creating an asymmetry of mass, charge and surface pressure. Subsequently, this asymmetry is dissipated by the re-organization of the lipid bilayer, leading to a transient increase in the permeability of the peptides across the lipid bilayer until an equilibrium is established between both sides of the bilayer. A similar occurrence of transient permeability and the subsequent appearance of resistance in the lipid bilayer following the action of melittin has been shown in phosphatidylcholine (PC) vesicles at a peptide/lipid ratio of 1/200 [40]. Consequently, experiments conducted at high peptide concentrations could be problematic when studying the interaction of peptides with lipid bilayers. In addition, the concentrations of melittin required to induce leakage [17,18,24,25,55,57] are in the sub-μM to low-μM range, and using much higher peptide concentrations might affect the relevance of the observed binding to the mechanism of membrane disruption. In a more recent SPR study, Aguilar and co-workers [62] re-investigated the interaction of melittin with DMPC at lower concentrations (0.125–12 μM). This study reported a concentration-dependent change in binding (resonance units or RU levels); however, it did not quantify the changes in binding affinity due to the reported poor fits for the 1:1 and two-state reaction binding models.

In this study, we carried out SPR experiments of the binding of melittin to 1-palmitoyl-2-oleoylphosphatidylcholine (POPC) membranes using three sets of concentration ranges that covered two orders of magnitude (0.04–8.0 µM), and also investigated the effect of ionic strength on this interaction. To our knowledge, this is the first study reporting extensive quantitative analysis of the concentration-dependent kinetics and binding affinity of melittin and how this might relate to the various stages of its mechanism of membrane binding and insertion.

## 2. Results

The POPC SUVs were deposited on the L1 sensor chip at a flow rate of 5 µL/min for 60 min, attaining an immobilization level up to a maximum of ~8400 RU. Following immobilization, multi-cycle or single-cycle kinetics experiments were conducted and the resulting sensorgram data were fitted to either the 1:1 Langmuir model or a two-state reaction model to estimate *k*_a_, *k*_d_ and *K*_D_ of the melittin–POPC interaction. As noted in Section 2, single-cycle kinetics experiments were used for the low-range and medium-range concentrations, while multi-cycle kinetics experiments were used for the high-range concentrations.

### 2.1. Effect of Analyte Concentration on Kinetics Analysis

One of the aims of this study was to investigate the effect of melittin (analyte) concentration on the apparent affinity of its interaction with POPC, the quality of fit to the data using different kinetic binding models and the subsequent reliability of the estimated binding constants. Multi-cycle kinetics (MCK) or single-cycle kinetics (SCK) experiments were carried out with three different ranges of analyte concentrations, referred to as high-range, mid-range and low-range. The analyte concentrations were: 0.5, 1, 2, 4 and 8 µM for the high-range; 0.3, 0.6, 0.9, 1.2 and 1.5 µM for the mid-range; and 0.04, 0.06, 0.08, 0.1 and 0.12 µM for the low-range.

**Figure 1:**
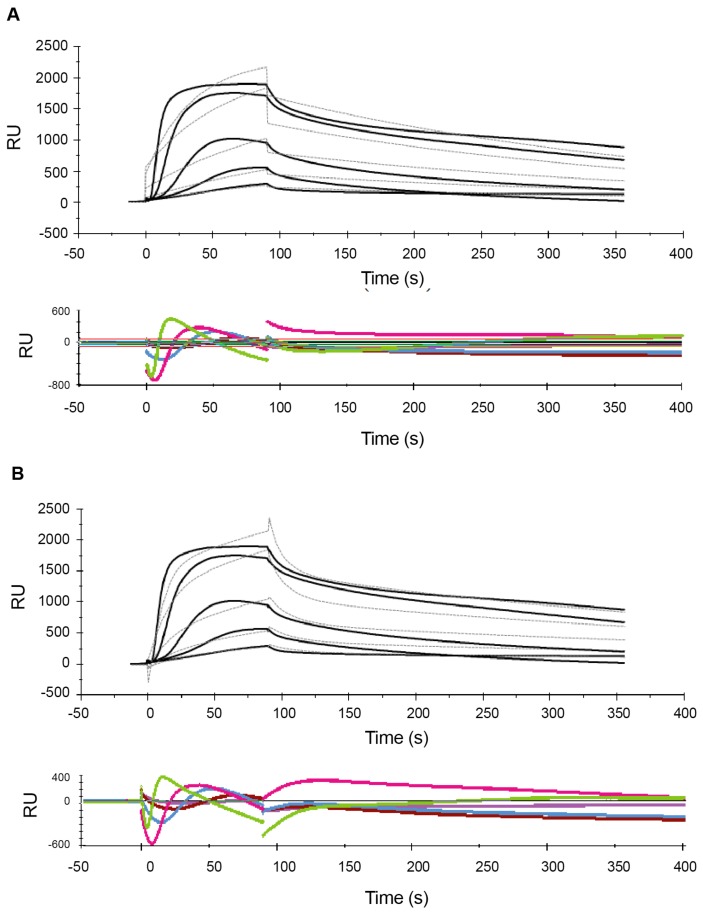
Sensorgrams of the melittin–POPC (1-palmitoyl-2-oleoyl-glycero-3-phosphocholine) interaction using multi-cycle kinetics for the high-range concentrations. Following immobilization of POPC liposomes (1 mM lipid) to a binding response of 8400 RU, increasing concentrations of melittin (0.5, 1, 2, 4 and 8 µM) were serially injected at a flow rate of 30 µL/min for 80 s with intermittent regeneration cycles with 10 mM glycine–HCl (pH 2.5). Binding kinetics analysis of the sensorgrams with a Langmuir 1:1 model (**A**) and a two-state reaction model (**B**) were conducted using BIAevaluation^®^ software. The estimated rate constants and affinity values, based on both these models, are listed in Table 1 and Table 2.

**Figure 2:**
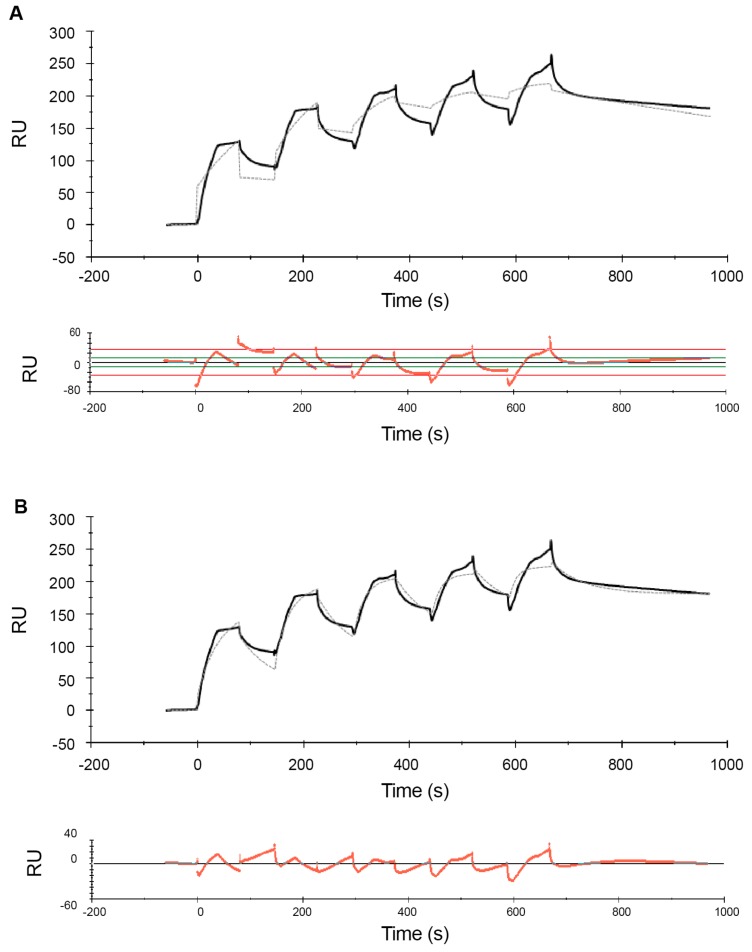
Sensorgrams of the melittin–POPC interaction using single-cycle kinetics for the mid-range concentrations. Following immobilization of POPC liposomes (1 mM lipid) to a binding response of ~4500 RU, increasing concentrations of melittin (0.3, 0.6, 0.9, 1.2 and 1.5 µM) were serially injected in a single-cycle at a flow rate of 30 µL/min for 80 s with a single regeneration step with 10 mM glycine–HCl (pH 2.5) at the end, i.e., after all five concentrations of melittin. Binding kinetic analysis of the sensorgrams with a Langmuir 1:1 model (**A**) and a two-state reaction model (**B**) were conducted using BIAevaluation^®^ software. The estimated rate constants and affinity values, based on both these models, are listed in Table 1 and Table 2.

**Figure 3:**
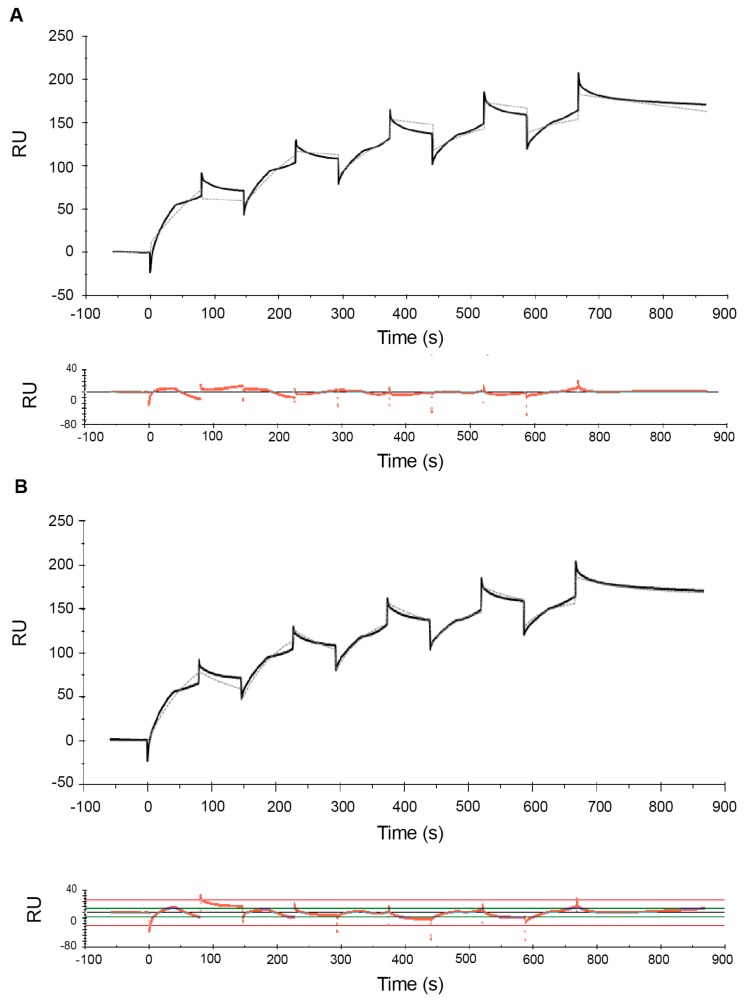
Sensorgrams of the melittin–POPC interaction using single-cycle kinetics for the low-range concentrations. Following immobilization of POPC liposomes (1 mM lipid) to a binding response of ~3500 RU, increasing concentrations of melittin (0.04, 0.06, 0.08, 0.1 and 0.12 µM) were serially injected in a single-cycle at a flow rate of 30 µL/min for 80 s with a single regeneration step with 10 mM glycine–HCl (pH 2.5) at the end, i.e., after all five concentrations of melittin. Binding kinetics analysis of the sensorgrams using a Langmuir 1:1 model (**A**) and a two-state reaction model (**B**) were conducted using BIAevaluation^®^ software. The estimated rate constants and affinity values, based on both these models, are listed in Table 1 and Table 2.

Figure 1 shows the sensorgrams obtained from MCK experiments with the high-range concentrations, while Figure 2 and Figure 3 show the sensorgrams from SCK experiments obtained with the mid- and low concentration ranges, respectively. For the high concentration range, MCK were used as attempts with SCK resulted in very high RU levels with the initial injections, which saturated the signal for subsequent additions. For all concentration ranges, data were fitted to a 1:1 Langmuir model and a two-state reaction model, and the corresponding residual plots are shown along with the fit of the model to the raw data in the sensorgrams. In addition to this, a steady-state fit with the maximum binding response at equilibrium for each analyte concentration was conducted for all the concentration ranges (see Figures S1–S3). Table 1 and Table 2 report the estimates of the kinetic parameters, resulting binding affinities, R_max_ as well as the Chi^2^ and *U*-values for the 1:1 Langmuir model and the two-state reaction model, respectively. Experiments were conducted three times with freshly prepared POPC liposomes each time, and the data is reported as mean ± SEM.

The 1:1 model assumes the formation of an analyte (A)-ligand (L) complex in a single step and with a 1:1 ratio, given by the reaction scheme

(1) $$A+L\;\begin{array}{c} k_{d}\\ ⇋\\ k_{a} \end{array}\;\mathrm{AL}$$

where *k*_a_ is the association rate constant for formation of AL, and *k*_d_ is the dissociation rate constant for complex AL. While this is one of the most commonly used kinetic binding model in SPR analysis, data from various studies indicates that the inherent assumptions of this model do not appropriately explain the complex mechanism involved in the binding of AMPs to membranes [65]. In contrast, the two-state reaction model assumes that the AL complex formed on the membrane surface undergoes a change (either a conformational change, a change in agglomeration or orientation) to form AL* and is given by the reaction scheme

(2) $$A+L\;\begin{array}{c} k_{d1}\\ ⇋\\ k_{a1} \end{array}\;\mathrm{AL}\;\begin{array}{c} k_{d2}\\ ⇋\\ k_{a2} \end{array}{\;\mathrm{AL}}^{*}$$

where *k*_a1_ is the association rate constant for formation of AL, *k*_d1_ is the dissociation rate constant for complex AL, *k*_a2_ is the association rate constant for conversion of AL to AL*, and *k*_d2_ is the dissociation rate constant for conversion of AL* to AL. The model assumes that the formation of AL* (and back) can only go via AL (i.e., A and L cannot form AL* and AL* cannot breakup without going through the state AL). It also assumes that the formation of AL follows first-order kinetics and that the rate of change is equal in both directions [74]. Note that it is not possible by SPR alone to determine the exact nature of the AL complex or determine the type of change that the complex undergoes between AL and AL*.

Comparison of the sensorgrams across the three concentration ranges used shows that one of the striking differences among the experiments at different concentration ranges were the R_max_ values. In the low- and mid concentration ranges, R_max_ levels are ~200 RU (Table 1 and Table 2). In contrast, for the high concentration range, R_max_ reached much higher levels (>2800 RU and >3400 RU for the 1:1 and two-state model, respectively). Interestingly, the difference in R_max_ values between mid- and low concentration ranges is substantially smaller to that observed in the high concentration range. This is observed in both the 1:1 and the two-state reaction models (Table 1 and Table 2). This is consistent with the widely accepted view that the binding of melittin to lipids is not based on the absolute concentration of the peptide, but rather it is determined by the peptide/lipid (P/L) ratio [26].

Comparison of the residual plots for the three concentration ranges, for both the 1:1 and two-state reaction models, shows that the fits were much better in the mid- and low concentration ranges than in the high concentration range. This is reflected quantitatively in the corresponding Chi^2^ and *U*-values (Table 1 and Table 2). Chi^2^ measures the average deviation of the experimental data from the model and is an overall measure of the “goodness of fit”, with values below 10% of R_max_ being considered as a good fit. *U*-values are a measure of the “uniqueness” of the predicted *K*_D_ value with a lower *U*-value indicating greater confidence in the predicted value. With the 1:1 model the Chi^2^ values range from 3% for the low concentration range to 7% for the high concentration range. With the two-state model the Chi^2^ values range from 2 to 4%. Similarly, the *U*-values are lower for the mid- and low concentration ranges than the high concentration range, indicating greater confidence in the estimated *K*_D_ values for mid- and low concentration ranges compared to high concentration range. The Chi^2^ and *U*-values are lower for the two-state model compared to the 1:1 model, indicating that the fit is much better for the former. This is consistent with observations from previous SPR studies of melittin by Aguilar and co-workers [62,65] whose comparison of the 1:1 model, a parallel model and the two-state model suggested that the fit for the latter is comparatively better.

Comparison of kinetic rate constants and the binding affinity from the different concentration ranges of melittin (analyte) used in this study indicate that these parameters are concentration dependent. This is true for both the 1:1 and the two-state model. While the absolute values of the kinetic rates and binding affinities are different between the models, the concentration-dependent trends are the same. Also, it is generally recommended to use a concentration range that the expected *K*_D_ value lies within the range of concentrations used to derive that *K*_D_ value. The *K*_D_ value, estimated as per the steady-state fit, for all three concentration ranges are within the analyte concentration range used; this is shown in the Supplementary Materials (Figures S1–S3). A steady state affinity measurement is based on the plot of responses at equilibrium states against the concentrations of analyte used in the assay. However, it is notable that the *K*_D_ values estimated using kinetic measurements (i.e., the *k*_a_ (on-rate) and *k*_d_ (off-rate) values), especially for the mid and low concentration ranges, were outside the analyte concentration range. This could be due to the fact of several reasons; in principle, the rate constants *k*_a_ and *k*_d_ and the *K*_D_ derived from these rate constants are independent of both analyte and ligand concentrations. Secondly, as can be noted in Table 1 and Table 2, the concentration-dependent effect on the change in affinity (*K*_D_) was higher due to the increasing on-rate (*k*_a_) than due to the observed decrease in the off-rate (*k*_d_). Other factors that may affect the *K*_D_, such as the pH, temperature etc., were kept constant across the experiments.

In both models, the association is slower in the high concentration range than in the low concentration range. For example, in the two-state model, the rate for the initial surface binding (*k*_a1_) was 2.1 × 10^5^ M^−1^·s^−1^ for the low concentration range, and it dropped to 4.1 × 10^4^ M^−1^·s^−1^ for the mid concentration range. The *k*_a1_ further decreased to 5.2 × 10^3^ M^−1^·s^−1^ for the high concentration range (Table 2). In other words, a 100-fold increase in concentration resulted in an approximately 100-fold decrease in the association rate. The association rates (*k*_a1_ and *k*_a2_) derived from the two-state reaction model also suggested that the initial binding to form the AL complex was much more rapid than the subsequent transition to AL*, and that *k*_a1_ was affected more by the concentration than *k*_a2._ Nevertheless_,_ in both models all association rates decreased with increasing peptides concentrations. This is inconsistent with previous findings reported by Aguilar and co-workers [62], where the association for a 0.125–1 µM concentration range was found to be slow, while rapid association was observed at higher concentrations (4–12 µM). However, this observation was based on a qualitative assessment of sensorgrams rather than a quantitative analysis, as the authors concluded that the latter was not possible due to the poor fit of their experimental data which may likely be due to the very high RU levels. In addition, the conclusion about the absence of binding at low concentrations was based on a comparison of the RU levels at low peptide concentrations (which were well below 200 RU) to the RU levels at high peptide concentrations (which were in the range of 3000–7000 RU). A semi-quantitative comparison of data with such different RU levels is likely less reliable than the analysis of binding kinetics rates derived from the sensorgrams.

Comparison of the dissociation rates from the different concentration ranges suggests that *k*_d_ might depend more on the number of analyte–ligand complexes formed (as given by R_max_ values) rather than the analyte concentration. In the 1:1 model this can be seen by comparing the *k*_d_ of the mid and low concentration ranges to the high concentration range. The *k*_d_ values from the mid and low concentration ranges were close to each other and both concentration ranges showed low RU levels. For the high concentration range, where the RU level was much higher, *k*_d_ deviated by an order of magnitude to that in the other two concentration ranges. A similar effect was seen for *k*_d1_ in the two-state model.

As a result of these concentration-dependent changes in the association and dissociation rates, the binding constant *K*_D_ differed by orders of magnitudes for the low, mid and high concentration ranges. For both binding kinetics models, *K*_D_ was in the µM (10^−6^) range for the high concentration range but shows nM (10^−9^) and sub-nM affinity for the low concentration range in the 1:1 and two-state reaction models, respectively.

### 2.2. Effects of NaCl on the Melittin–POPC Interaction

**Figure 4:**
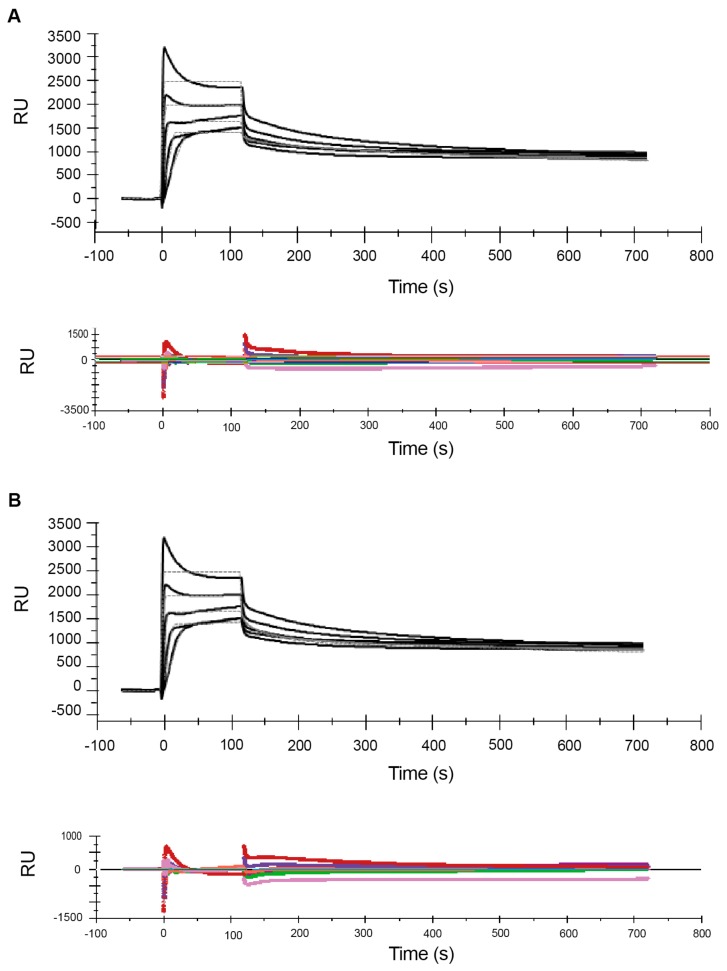
Effect of sodium chloride on the association phase of the melittin–POPC interaction. Following immobilization of POPC liposomes (1 mM lipid), increasing concentrations of melittin (2, 4, 6, 8 and 10 µM) were serially injected with intermittent regeneration cycles with 10 mM glycine–HCl (pH: 2.5) (**A**—multi-cycle) and single regeneration using the same buffer at the end of all cycles (**B**—single-cycle). The estimated rate constants and affinity values, based on both these models, are listed in Table 1 and Table 2.

**Figure 5:**
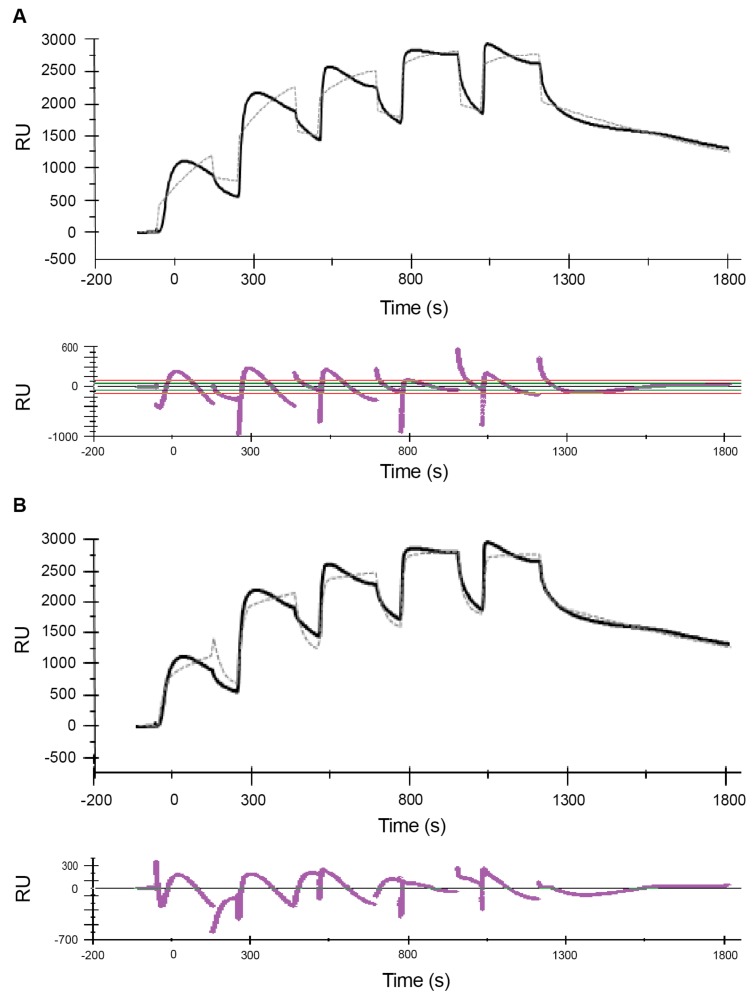
Effect of sodium chloride on melittin–POPC interaction—single-cycle kinetics. Following immobilization of POPC liposomes (1 mM lipid), increasing concentrations of melittin (0.5, 1, 2, 4 and 8 µM), made up in running buffer containing 0.15 M NaCl, were serially injected, following a single-cycle kinetic approach. The sensorgrams were fitted to a 1:1 Langmuir model (**A**) and two-state reaction model (**B**) using BIAevaluation^®^ software. The estimated rate constants and affinity values, based on both these models, are listed in Table 1 and Table 2.

Ionic strength is known to affect the biological activity of AMPs [75] including the oligomerisation of melittin in solution [29,70,71]. To assess the effect of NaCl on the binding of melittin to POPC, experiments with melittin in the high concentration range (0.5 to 8 µM) were conducted in the presence of 0.15 M NaCl in the running/analyte buffer. Figure 4 and Figure 5 show the sensorgrams for MCK and SCK for the melittin–POPC interaction in the presence of 0.15 M NaCl, fitted to 1:1 and two-state reaction models, respectively. Comparison of this figure with the sensorgrams obtained in the absence of NaCl (Figure 1) suggests that addition of NaCl results in a decrease in signal response during the association phase for almost all of the concentrations. Consequently, the sensorgrams did not have the desired curvature optimal for performing fitting analysis. However, the curve-fitting step was conducted for experiments involving the use of NaCl and the estimates are reported, but no major conclusions were made using the estimates. Comparison of the R_max_ levels in Table 1 and Table 2 shows that the drop from 2841 RU to 2168 for the 1:1 model and from 3488 to 2725 RU in the two-state model, corresponds to ~20%–25% reduction in R_max._ This decrease in signal response resulted in only a small decrease in the association rate (*k*_a_) in the 1:1 model (Table 1). In the two-state model, it was *k*_a*2*_ that was reduced, while *k*_a1_ slightly increased in the presence of NaCl. In both models, the dissociation rate (*k*_d_ for the 1:1 model and *k*_d1_ for the two-state model) was also reduced, more so than the reduction in *k*_a_. As a result, there was an overall small increase in affinity upon addition of NaCl.

## 3. Discussion

This study aimed to quantify the effect of peptide concentration on the kinetics and affinity of melittin binding to POPC bilayers. The kinetics of the melittin–POPC interaction was studied at three different concentration ranges spanning two orders of magnitude. To understand the mechanism of melittin (and other AMPs), it is not so much the actual concentration of peptide present that is important but the relative amounts of peptide and lipid, i.e., the P/L ratio. The analyte concentrations used in this study correspond to the following P/L ratios: high range concentration (0.5–8 µM) is a P/L of ~1/2000 to 1/125; mid range concentration (0.3–1.5 µM) is a P/L of ~1/3330 to 1/660; low range concentration (0.04–0.12 µM) is a P/L of ~1/20,000 to 1/8300.

As noted in the introduction, there have been previous studies reporting the binding affinity of melittin for phospholipid bilayers. When comparing binding affinities for peptide–membrane systems, it is important to consider the technique or assay used as this often dictates the theoretical model used to analyse the data. In SPR, *K*_D_ is calculated using binding kinetics rates and/or Langmuir adsorption isotherms or related binding models. In contrast, data from ITC, NMR or filtration experiments of peptide–membrane systems can be analysed using a surface partition equilibrium model. In this case, a partition coefficient *K*_p_, rather than *K*_D_, is calculated. *K*_p_ is given by the ratio of peptides bound to the membrane and peptides remaining in solution, while electrostatic effects are corrected for by using the Gouy–Chapman theory [76]. A direct comparison of the raw data between these types of experiments or analysis using both models is often not possible. In both cases, the free energy associated with the binding affinity is given by ΔG = −RT ln(*K*), where R is the gas constant, T is the temperature in Kelvin and *K* is either *K*_p_ or 1/*K*_D_. Nevertheless, the underlying assumptions in these models are different and affinities for the same system under the same conditions can vary [77]. In the following discussion, the ΔG = −RT ln(*K*) relation is used for comparison of data from ITC and NMR experiments from previous studies and the SPR data from this study.

In one of the earliest studies of melittin binding to membranes, Vogel [35] used circular dichroism (CD) and fluorescence spectroscopy to estimate the binding affinity of melittin to DMPC vesicles. The lipid and peptide concentration used in that study correspond to P/L ratios between 1/10 to 1/250 which is comparable to the upper end of the high concentration range used in this study. The *K*_D_ from the CD titration experiments was calculated using a binding isotherm and estimated to be 2 µM which is in very good agreement with the binding affinities of 1.2 µM and 1.7 µM of our experiments. Allende et al. [55,59] used an ultrafiltration assay to study the interaction of melittin with membranes of a wide range of different lipid compositions including egg phosphatidylcholine (EPC) and DOPC vesicles. The P/L ratios ranged from 1/300 to 1/600, corresponding to our high concentration ranges. The *K*_p_ values for EPC and DOPC were ~3.6 µM, slightly higher but still in good agreement with our results. In two NMR studies, Beschiaschvili et al. [60,78] estimated the binding affinity of melittin to POPC vesicles using P/L ratios from ~1/600 to 1/1400. This corresponds to the lower part of the high range and upper part of the mid range from our experiments. The *K*_p_ was estimated between 1.5 to 2 µM. This agrees with our *K*_D_ values for the high concentration range but not for the values in the mid concentration range, which are estimated to be much lower (sub-µM and nM range). However, a direct comparison of the *K*_D_ values from our SPR experiments to the *K*_p_ values from the NMR is complicated by the fact that, based on our findings, the concentration range used in the NMR experiments is where the effect of concentration is the largest. Another difference is that in the aforementioned studies the size of the vesicles used ranges from 1–10 µM which is 20–200 times larger than the vesicles used in this study. This difference in size implies differences in the curvature of the membrane surface, which can affect the peptide-lipid interaction.

The only other SPR study that used quantitative analysis to report *K*_D_ values of melittin binding to PC-only membranes is that by Lee et al. [64], where the binding of melittin to DMPC was studied. The *K*_D_ value was estimated to be 33 µM for P/L ratios between 1/3 and 1/100 which is higher than the highest concentration used in our study. This could be the result of the very high P/L ratio or the fact that supported lipid monolayers formed on an HPA chip instead of the intact bilayer vesicles used in this study. In our experiments, we assumed that the vesicles (ligand) stay intact after immobilization [79]. The tighter lipid packing of a supported bilayer compared to SUVs might cause slightly reduced binding [80].

In another SPR study, Papo et al. [68] quantified the kinetics and affinity of binding of melittin to POPC/chol (10:1 w/w) vesicles using P/L ratios between ~1/1600 and ~1/3300. This corresponds roughly to the low range concentrations used in our study. Data were analysed using a two-state model, and the *K*_D_ estimated based on this model was 1.6 µM. This is significantly lower than the *K*_D_ determined in the sub-nM range for our low concentration. While the study by Allende et al. [55] showed that cholesterol can lower the binding affinity by 1–2 orders of magnitude, there is still a large difference between the *K*_D_ reported by Papo et al. and our estimates in this study. We note, however, that the value of *k_a_*_1_ reported by Papo et al. was very low and could possibly be below the acceptable detection limit of the Biacore X instrument used in their experiments. We speculate this based on the acceptable limits for the kinetic binding constants for the Biacore T200 instrument used in this study which is a more sensitive instrument with a wider detection limit compared to the Biacore X instrument.

Overall, the results of our SPR experiments for the high concentration range are in good agreement with the binding affinities from earlier studies. Combined with the fact that our estimates of the binding affinities in the mid- and low-range concentrations are more reliable, it increases our confidence in the predicted effect of concentration on the kinetics and binding affinity in the mid- and low concentration ranges. We can now interpret our finding that the apparent binding affinity of melittin for POPC decreases with increasing P/L ratio in the context of what is known about the mechanism of action of melittin.

As mentioned in the introduction, a number of studies have shown that melittin can orient itself into different orientations with respect to the bilayer surface: in a parallel orientation, where the peptide lies flat on the membrane surface, and a perpendicular orientation where the peptide inserts into the hydrophobic core region of the bilayer. The parallel orientation is often referred to as the ‘inactive’ state, as there is evidence suggesting that this orientation is non-pore forming and non-lytic. In contrast, the perpendicular orientation is the ‘active’ state that results in the formation of the pores [26,81,82]. In the original two-state model [41] the peptide binds to the surface and then transitions from the parallel to the perpendicular orientation. In an alternative model, parallel surface binding and direct insertion are competing processes [26]. Independent of the mechanism, there is evidence that suggests that the most important factor controlling the amount of peptides found in the parallel or perpendicular orientation is the P/L ratio [26,81]. Yang et al. [81] used oriented CD spectroscopy and neutron scattering to demonstrate that at melittin concentrations corresponding to a P/L ratio of ~1/40, 85% of peptides were orientated parallel to the membrane surface. At peptide concentrations corresponding to a P/L ratio of ~1/15, 68% of peptides were orientated perpendicular to the membrane. Another study demonstrated that at higher P/L ratio, melittin inserts perpendicularly into the hydrophobic core of the bilayer, to translocate and redistribute on either side of the bilayer [54]. This study suggests that the redistribution of melittin on either side of the bilayer at a higher P/L ratio (~1/100) occurs due to the formation of transient but not stable pores, and that the latter occur only above a critical P/L ratio (~1/45). Interestingly, the formation of such transient pores in phospholipid bilayers by the action of melittin has also been observed at concentrations as low as a P/L of 1/5000. For SUVs, this P/L ratio corresponds to the low- and mid-range concentrations used in this study. Similarly, the existence of transient pores induced by melittin and the subsequent ‘re-sealing’ has been shown in bacterial membranes [72]. Based on these findings, a variant of the binding kinetics model of the classical carpet model has been proposed, in which permeabilisation of membranes by cationic amphipathic peptides is transient and stochastic on the scale of a single vesicle or bacteria [40,72,83,84]. Peptides accumulate on the bilayer surface, causing an asymmetry in peptide concentration across the bilayer, i.e., an asymmetry in mass, charge and surface pressure on the bilayer. Subsequently, this asymmetry is abolished by a set of stochastic membrane re-organization events, resulting in the influx of peptide molecules across the bilayer until equilibrium in concentration is attained. Finally, simulations show that while the energy barrier for the reorientation of single peptide is large, the barrier is reduced by a higher P/L as well as by the presence of transient water-filled pores in the membrane [45,85].

Based on the above findings, we can assume the following conditions at the low- and mid concentration ranges used in our experiments: the vast majority of peptides are found in parallel orientation and no stable pores are present, but transient pores might stochastically form and ‘re-seal’. This means that at a low P/L ratio, any additional increase in peptide concentration results in an accumulation of peptides on the membrane surface creating an accumulation of positive charge, leading to an increase in the repulsion for subsequent peptide binding. This could explain the continuous decrease in the association rates (*k*_a1_ and *k*_a_) with increasing concentration of melittin observed in this study. The largest effect of concentration on K_D_ occurs when going from the mid- to the high concentration range, which goes from supra-nM to µM range. The upper range of the high concentration range in this study reaches a P/L ratio where the presence of peptides found in the perpendicular orientation is increasingly likely. This is consistent with the fact that concentration ranges used in this study were relevant to the concentration of melittin required to induce leakage [17,18,24,25,55,57] and the minimum inhibitory concentration of melittin on bacteria.

Our experiments also showed that binding kinetics parameters were also affected by the choice of binding model. At all concentrations, a two-state reaction model gives the best fit to the data, suggesting that it is better at describing the process of binding than a 1:1 model. At first this appears to confirm the classical two-state model of melittin binding and pore formation [41] which involves the transition from a parallel to a perpendicular orientation, excluding the model where parallel adsorption and perpendicular insertion are described as competing processes [26]. However, our findings only confirm that a two-state model provides a better description of binding than the 1:1 model, but cannot exclude the parallel–perpendicular competition model [26]. This would only be possible by a direct comparison of data fitted to both of these models. We note that the conventional binding models used in this study do not account for any electrostatic effects. The CD spectroscopy experiments by Beschiaschvili et al. [60] demonstrated that melittin binding to lipids (SUVs) decreased as the membrane surface charge was neutralized by previously bound melittin. This effect was observed to be higher in zwitterionic lipids (POPC) than hybrid SUVs (80/20 POPC/POPG). If taken into account, the influence of electrostatic effects could potentially correspond to the transition from positive to negative cooperativity in melittin–membrane interactions which, to the best of our knowledge, cannot be qualitatively assessed by SPR.

In addition to the effect of peptide concentration on the kinetics and affinity of binding, we also investigated the effect of physiological ionic strength (0.15 M NaCl) on the interaction of melittin with POPC bilayers. At the pH, ionic strengths and peptide concentrations used in this study, melittin is be expected to remain in its monomeric form [28,29,69,70,71]. In the presence of NaCl, an apparent decrease in the signal response during the association phase was observed in both single and multi-cycle kinetics experiments, leading to a somewhat reduced association rate (*k_a_*). This could be caused by a screening effect of the NaCl between the cationic peptide and the zwitterionic membrane. Another reason could be that the binding of Na^+^ ions to the membrane surface changes the electrostatics of the water-lipid interfacial region. Several studies have shown that Na^+^ ions can bind to phospholipid bilayers, where they interact with the phosphate and carbonyl oxygen of the lipid headgroups [86,87]. The binding of Na^+^ shields the negative charge of the phosphate group and, thus, indirectly increases the electrostatic charge of the membrane (as there are now more unbound positively charged choline groups than negatively charged phosphate groups). As the initial association is likely driven by electrostatics, this could account for the reduced *k*_a_ in the presence of NaCl. Further characterisation of the melittin–POPC interaction with a range of NaCl concentrations will be needed to better understand this effect. Also, future studies should focus on the use of complex cell membrane models (e.g., hybrid lipid systems or virus-like particles derived from mammalian cell models) that could enable interaction studies in a more physiologically relevant context.

## 4. Materials and Methods

### 4.1. Materials

The POPC (1-palmitoyl-2-oleoyl-glycero-3-phosphocholine) was purchased from Avanti Polar Lipids (Birmingham, USA) through Sigma–Aldrich (Castle Hill, Australia). Mechanical extrusion for the preparation of lipid vesicles was carried out using an extruder from Avanti Polar Lipids (Birmingham, USA). Melittin was purchased from AnaSpec (Fremont, USA). Reagents for buffer solutions, glycine-HCl and CHAPS (3-((3-cholamidopropyl) dimethylammonio)-1-propanesulfonate) solutions were purchased from Sigma–Aldrich (Castle Hill, Australia). Running buffer for SPR experiments consisted of 0.05 M HEPES (Sigma–Aldrich, Australia) at a pH of 7.4 and also included 150 mM NaCl for experiments investigating the effect of ionic strength. The SPR experiments were conducted in a BiacoreT200 instrument using a L1 sensor chip S series (GE Healthcare Life Sciences, Paramatta, Australia).

### 4.2. Liposome Preparation

The POPC vesicles at a 1.0 mM lipid concentration were used for all experiments in this study and were prepared as follows. First, the required amount of POPC was weighed in a glass vial, dissolved in chloroform and dried under a narrow jet of nitrogen gas. Complete dehydration of the lipid film was ensured by placing the vial in a vacuum desiccator attached to a pump for at least 12 h. The resulting lipid film was rehydrated with running buffer to a final lipid concentration of 1.0 mM. The solution was vortexed for 1 h and allowed to stand for at least 2 h or to a maximum overnight period to enable osmotic swelling. A uniform population of small unilamellar vesicles (SUVs) was obtained by mechanical extrusion through a polycarbonate filter with pore size of ~50 nm for at least 21 times. This approach was previously shown to be effective in attaining a higher degree of homogeneity and reproducibility in nanosizing liposomes compared to other commonly used approaches for generating SUVs [88].

### 4.3. Surface Plasmon Resonance Experiments

#### 4.3.1. Immobilization of POPC Vesicles on a L1 Chip

The L1 sensor chip was equilibrated at room temperature and then docked into a BiacoreT200 instrument. After priming the system with running buffer, a manual run was set-up for three consecutive injections with running buffer containing 20 mM CHAPS at a flow rate of 30 µL/min. This washing step was conducted in every subsequent use of the chip before and after the binding experiments. The POPC SUVs of ~50 nm diameter at a 1.0 mM lipid concentration were then steadily immobilized on to the L1 chip surface through a manual run at a flow rate of 5 µL/min. The target immobilization levels varied from ~4000 to 8000 RU, based on the concentration of melittin used in the binding assay. Following liposome immobilization, a short pulse with running buffer was injected at a flow rate of 2 µL/min for 5 min to ensure the stability of the baseline in the flow cell containing the immobilized vesicles.

#### 4.3.2. Binding experiments

Melittin solutions at the desired range of concentrations were prepared in running buffer by dilution from a stock solution (2.2 mM). Melittin solutions were also prepared in a running buffer containing 0.15 M NaCl, the POPC SUVs were prepared in a running buffer without NaCl, while all other experimental conditions were the maintained as same as in the experiments conducted in the absence of NaCl. The kinetics of the melittin–POPC interactions were estimated using either single or multi-cycle kinetics. Melittin solutions were serially injected at increasing concentrations at 30 µL/min, with (multi-cycle) or without (single-cycle) intermittent regeneration cycles among various analyte injections, using 10 mM glycine–HCl, pH 2.5.

In a single-cycle kinetics experiment increasing concentrations of analyte are serially injected with a single dissociation phase after the injection of the highest concentration. It should be noted that, although this approach includes only a single dissociation phase at the end, there is a short pulse of running buffer after each analyte injection period during when some dissociation can be noted. Depending on the rate of dissociation, it is possible that some analyte remains bound to the immobilized ligands before the next injection. However, the fitting models used for the analysis of single-cycle kinetic experiments account for the varying concentrations of analyte injections in time and different amounts of preformed ligand–analyte complexes on the surface [89]. The fact that in a single-cycle kinetic experiment there is no intermittent regeneration cycles, it can be advantageous as it prevents the detrimental effects of a strong acidic regeneration buffer on the ligand. Single-cycle and multi-cycle approaches differ in their experimental procedure but have been shown to yield comparable results. Nevertheless, the single-cycle approach can be challenging for high analyte concentrations, because the number of ligand–analyte complexes being formed after initial analyte injections can substantially impair analyte binding in subsequent injections. Therefore, a multi-cycle approach, which includes separate association, dissociation and regeneration phase for each analyte injection, is used only for the high-range analyte concentration in this study.

### 4.4. Curve Fitting

All sensorgrams obtained from single- and multi-cycle kinetic analyses were fitted using either the 1:1 Langmuir model or the two-state reaction model available in the BIAevaluation^®^ software version 1.0 (GE Healthcare Life Sciences, Paramatta, Australia) to estimate *k*_a_, *k*_d_ and *K*_D_. The curve fitting procedure is an iterative numerical process that determines the best fit of experimental data to the set of equations specific for a binding kinetics model (e.g., the 1:1 Langmuir or the two-state reaction model) thus defining the interaction and yielding the kinetic rate constants (*k*_a_ and *k*_d_) from which the binding constants (*K*_D_) can be determined. The “goodness of fit” for the fitted curve is given by Chi^2^. In addition, residual plots show the magnitude of the deviation (in units of RU) of the experimental data from the fitted data as a function of time. The experimental data are shown as solid lines and the fitted data as dotted lines in all of the sensorgrams. The quality of fit and reliability of the estimated binding parameters were assessed by the Chi^2^ and *U*-values (only for the Langmuir 1:1 model), respectively.

## Figures and Tables

**Table 1:** Kinetic and data-fitting parameters for melittin–POPC interactions based on a 1:1 Langmuir model. Data were measured at the three concentration ranges and obtained from fitting sensorgrams to a 1:1 Langmuir model to obtain estimates of association (*k*_a_) and dissociation rates (*k*_d_) as well as binding constants (*K*_D_), R_max_, Chi2 and *U* values. Uncertainties are given as mean ± SEM obtained from the curve fitting.

Analyte (Melittin) Concentration (µM)	*k*_a_ (M^−1^·s^−1^)	*k*_d_ (M^−1^·s^−1^)	*K*_D_ (M)	R_max_ (RU)	Chi^2^ (RU)	*U*-Value
0.5–8.0 (high-range)	2.2 ± 0.1 × 10^3^	2.7 ± 0.1 × 10^−3^	1.2 ± 0.1 × 10^−6^	2841 ± 24	192	12
0.5–8.0 (high-range) +0.15 M NaCl	1.1 ± 0.3 × 10^3^	8.2 ± 1.1 × 10^−4^	0.6 ± 0.3 × 10^−6^	2168 ± 28	198	12
0.3–1.5 (mid-range)	1.6 ± 0.2 × 10^4^	7.4 ± 0.2 × 10^−4^	4.1 ± 0.1 × 10^−8^	216 ± 11	12	9
0.04–0.12 (low-range)	1.2 ± 0.3 × 10^5^	5.7 ± 0.8 × 10^−4^	4.7 ± 0.6 × 10^−9^	197 ± 8	7	5

**Table 2:** Kinetic and data-fitting parameters for melittin–POPC interactions based on a two-state reaction model. Data were measured at the three concentration ranges and obtained from fitting sensorgrams to a two-state reaction model to obtain estimates of association (*k*_a1_ and *k*_a2_) and dissociation rates (*k*_d1_ and *k*_d2_) as well as binding constants (*K*_D_), R_max_ and Chi2 values. Uncertainties are given as mean ± SEM obtained from the curve fitting.

Analyte (Melittin) Concentration (µM)	*k*_a1_ (M^−1^·s^−1^)	*k*_d1_ (s^−1^)	*k*_a2_ (M^−1^·s^−1^)	*k*_d2_ (s^−1^)	*K*_D_ (M)	R_max_ (RU)	Chi^2^ (RU)
0.5–8.0 (high-range)	5.2 ± 0.1 ×10^3^	4.7 ± 0.2 ×10^−2^	1.3 ± 0.01 ×10^−2^	3.1 ± 0.1 ×10^−3^	1.7 ± 0.2 ×10^−6^	3488 ± 48	134
0.5–8.0 (high-range) +0.15 M NaCl	2.2 ± 0.6 ×10^4^	5.8 ± 1.2 ×10^−2^	3.8 ± 1.1 ×10^−3^	8.5 ± 0.7 ×10^−4^	0.5 ± 0.2 ×10^−6^	2725 ± 18	122
0.3–1.5 (mid-range)	4.6 ± 0.2 ×10^4^	1.3 ± 0.1 ×10^−2^	3.4 ± 0.3 ×10^−3^	1.2 ± 0.3 ×10^−6^	1.5 ± 0.2 ×10^−11^	243 ± 13	7
0.04–0.12 (low-range)	2.1 ± 0.1 ×10^5^	7.1 ± 0.1 ×10^−3^	5.2 ± 0.4 ×10^−3^	2.2 ± 0.8 ×10^−6^	1.5 ± 0.3 ×10^−11^	200 ± 9	3

## Supplementary Materials

Supplementary Materials can be found at https://www.mdpi.com/1422-0067/21/3/746/s1.
