# Supplementary material for: Revisiting the Interaction of Melittin with Phospholipid Bilayers: The Effects of Concentration and Ionic Strength
Source: Int J Mol Sci. 2020 Jan 23;21(3):746. doi: 10.3390/ijms21030746 (PMC7037773; doi:10.3390/ijms21030746)
Supplement: Supplementary file 1 [file ijms-21-00746-s001.pdf]

Revisiting the interaction of melittin with phospholipid bilayers: the effects of concentration and ionic strength

Supplementary Information

Thiru Sabapathy [1], Evelyne Deplazes [1,2,†] and Ricardo L. Mancera [1,†,*]

[1] School of Pharmacy and Biomedical Sciences, Curtin Health Innovation Research Institute, Curtin University, GPO Box U1987, Perth WA 6845, Australia
[2] School of Life Sciences, University of Technology Sydney, Ultimo NSW 2007 Australia
* Correspondence: R.Mancera@curtin.edu.au
† co- corresponding authors.

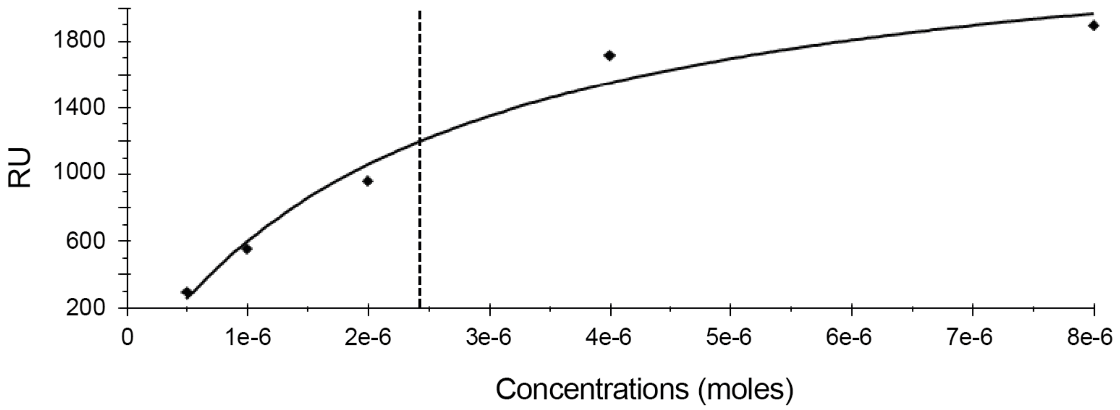

Figure S1 – Steady affinity curve for melittin:POPC interaction derived from multi-cycle kinetics plot– ‘high’ concentration range.

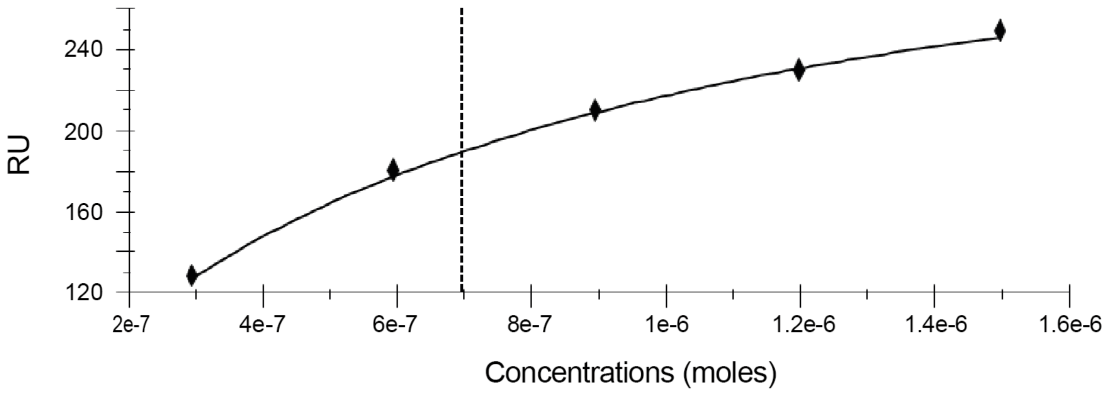

Figure S2 – Steady affinity curve for melittin:POPC interaction derived from single-cycle kinetics plot – ‘mid’ concentration range

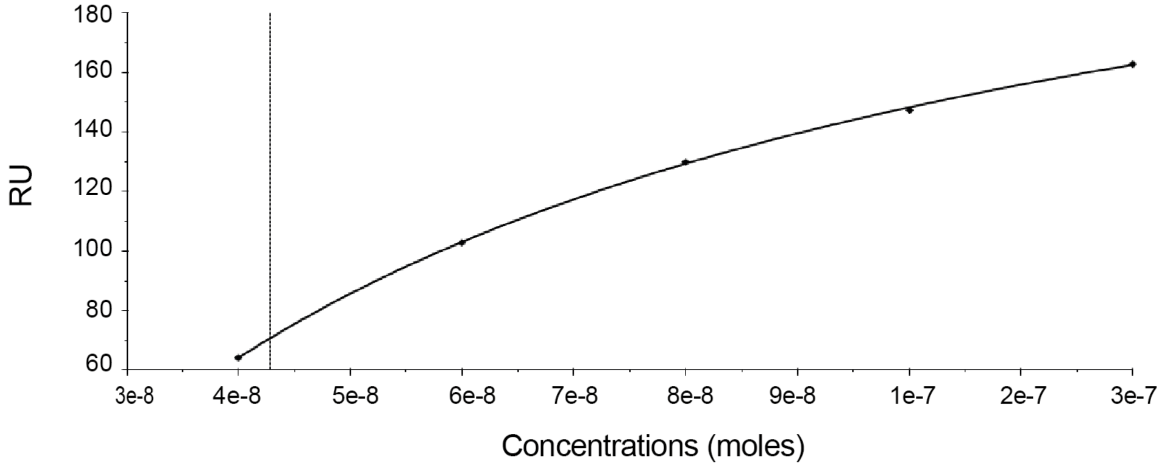

Figure S3 – Steady affinity curve for melittin:POPC interaction derived from single-cycle kinetics plot– ‘low’ concentration range.
